# Brain Mechanisms during Course of Anesthesia: What We Know from EEG Changes during Induction and Recovery

**DOI:** 10.3389/fnsys.2017.00039

**Published:** 2017-05-29

**Authors:** Satoshi Hagihira

**Affiliations:** ^1^Department of Anesthesiology, Kansai Medical UniversityOsaka, Japan; ^2^Department of Anesthesiology and Intensive Care Medicine, Osaka University Graduate School of MedicineOsaka, Japan

**Keywords:** electroencephalogram, GABA_A_ receptor, propofol, volatile anesthetic, consciousness

## 1. Introduction

The mechanism of anesthesia remains unclear. We do understand, however, that the *GABA*_A_ receptor, the NMDA receptor, and two-pore-domain *K*^+^ channels are the main targets of anesthetics (Franks, 2008), and several other receptors and ion channels are also influenced in different ways by anesthetics (Alkire et al., 2008). Propofol, barboturates, and volatile anesthetics, all potentiate *GABA*_A_ receptor. During isoflurane, sevoflurane, and propofol anesthesia similar raw electroencephalogram (EEG) pattern sequences are observed. By contrast, ketamine, nitrous oxide, and xenon, which antagonize the NMDA receptor are characterized by patterns distinctive for each agent. My discussion here focuses on anesthetics that potentiate the *GABA*_A_ receptor. Data in this experiment were obtained from Fp_1_–A_1_, or Fpz–At_1_, a hemi-frontal lead of the type commonly used with EEG-based anesthesia monitors.

The relation between consciousness and EEG patterns has been extensively studied, and it is now well-known that EEG changes correlate well with anesthetic effect. Meanwhile, anesthesia is usually considered to be a reversible phenomenon. The question is, then, do the changes in the brain anesthesia mirror each other during induction and recovery, or are they independent phenomena? Here, based mainly on EEG evidence gathered during anesthesia, I will discuss this question.

## 2. Are induction and recovery mirror processes or independent phenomenon?

In previous studies, raw EEGs were recorded throughout periods of anesthesia maintained by isoflurane (*N* = 32) (Hagihira et al., 2002) and propofol (*N* = 25) (Kang et al., 2017), and some conclusions were drawn from the data. When volatile anesthetic is used, rapid induction by thiopental or propofol is common. As a result, comparison of raw EEGs during induction and recovery is confounded by other agents. On the other hand, it is possible to slowly induce propofol anesthesia without other agents. When this is done, as presented in the following section, pattern sequences of raw EEG during induction and during recovery, for propofol, at least, show symmetry. In a study of volunteers, Iwakiri et al. (2005) have shown that effect-site concentration (Ce) of propofol at loss of consciousness is virtually identical to that at recovery of consciousness. Doufas et al. have also shown the same result (Doufas et al., 2004). Here, Ce is a virtual concentration that represent the drug effect by reckoning the drug concentration at transfer from blood to the effect site (brain) and vice versa. Evidence from observed EEG changes and Ce findings, show induction and recovery from propofol anesthesia to apparently be symmetrical processes based on a reversible mechanism.

Data in the Iwakiri and Doufas studies were derived from volunteers who didn't undergo surgical procedures: no other factors confounded observation of anesthetic effects. In usual clinical practice, however, wound pain and the use of analgesics to counteract noxious stimuli are likely to change raw EEG patterns (Hagihira et al., 2004). Thus, when a patient emerges from anesthesia in a clinical setting, other factors usually complicate the recovry process.

Recently, Ku et al. (2011) evaluating symbolic transfer entropy (STE) (Staniek and Lehnertz, 2008) which is based on information theory Schreiber (2000), reported that during induction of propofol anesthesia, feedback connections from the frontal region to the parietal region decreased and became equal to feed-forward connections from the parietal region to the frontal region, and it became predominant after recovery from anesthesia. This finding provides further evidence that induction and recovery mirror each other.

Meanwhile, from a murine model, Kelz et al. (2008) have reported, without affecting induction, that selective orexin-1 receptor antagonist delays emergence from isoflurane or sevoflurane anesthesia. Orexinergic neurons are known to play a critical role in promotion and maintenance of wakefulness, and Chemelli et al. (1999) have demonstrated that orexin knockout mice are phenotypically quite similar to human narcolepsy patients. To date, there have been no reports on the effects of orexin antagonist on recovery from anesthesia in humans. Further study is needed to clarify this.

## 3. EEG during slow induction of anesthesia

**Figure 1:**
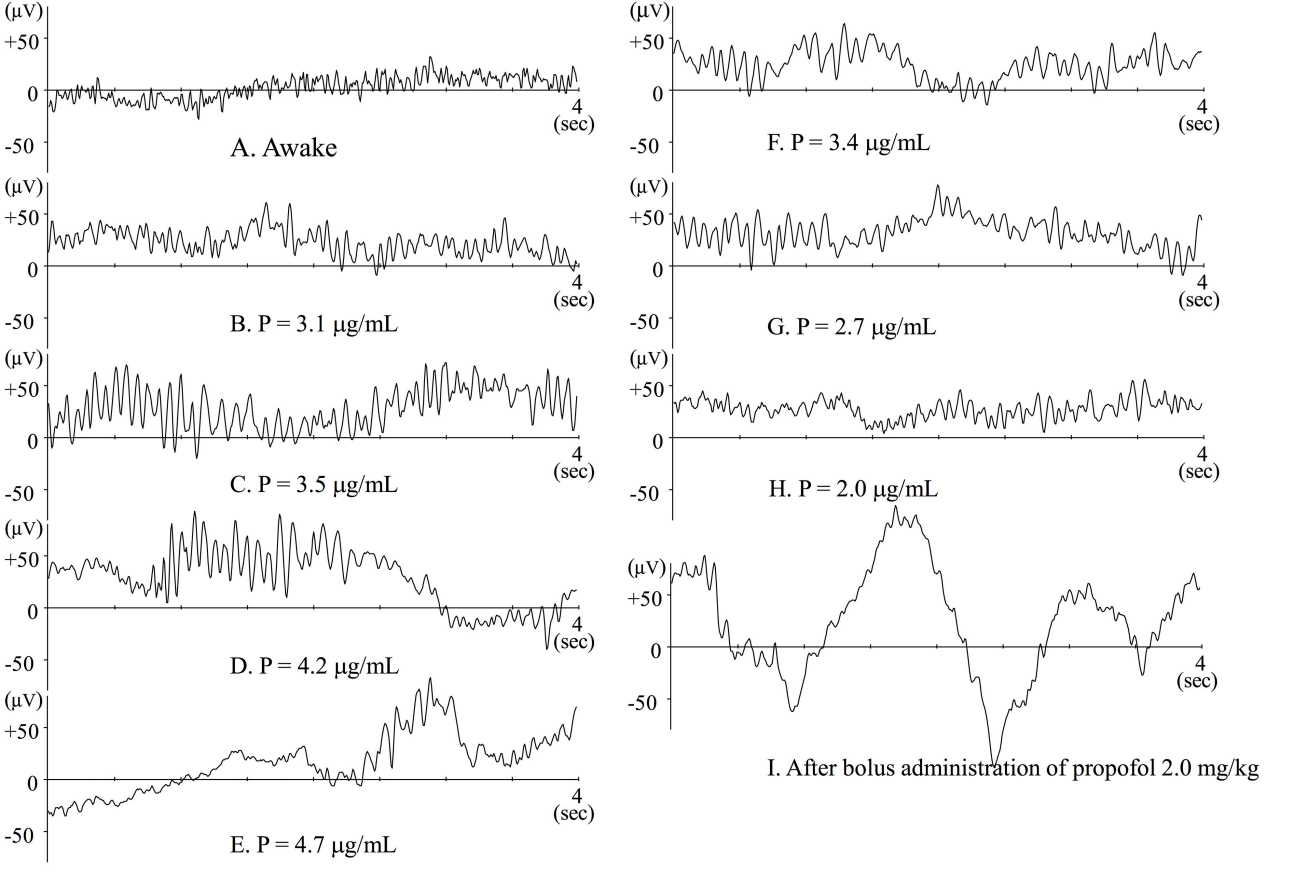
**Raw EEGs during slow induction of propofol anesthesia (A–E)**, and during recovery from propofol anesthesia **(F–H)**. **(A–H)** Raw EEGs obtained from the same patient (44-year-old female), and P indicates the effect-site concentration of propofol. **(I)** Raw EEG obtained from a 64-year-old female patient just after bolus administration of propofol 2 mg · kg^−1^. At that point, BIS™ value was 27.8.

Figure 1 showed typical raw EEG changes when anesthesia is gradually induced with propofol (Kang et al., 2017), and such changes were well-documented previously (Sloan, 1998; Bennett et al., 2009). When blood (and consequently effect-site) concentration of propofol slowly increases, patients are gradually sedated and finally lose responsiveness. When awake or during light sedation, EEG shows quite small and high frequency (Figure 1A). Electromyogram (EMG) contamination is often observed at this level. Just after loss of response, fast waves with small amplitude still predominate, but their frequency is slower than during wakefulness. Alpha power is quite small at this level (Figure 1B). As propofol Ce increases, EEG frequency slows and EEG amplitude becomes larger, whereupon alpha waves becomes dominant (Figure 1C). This waxing and weaning spindle wave pattern is observed during stage II of slow wave sleep. During sleep, spindles emerge only transiently, but are observed continuously during anesthesia induced by propofol or volatile anesthetics. When propofol Ce increases further, alpha waves become smaller and theta and delta waves become dominant (Figure 1D), eventually, the burst-suppression pattern emerges (Figure 1E).

Using EEG combined with simultaneous intracellular recordings, Steriade et al. (1993b,a) have extensively investigated the mechanism that generate spindles. They found that the spindle rhythm is generated in the thalamic reticular nuclei (RE) and thalamo-cortico-thalamic circuits, the EEG rhythm being determined by the membrane potential of thalamo-cortical relay (TC) neurons. Nuñez et al. (1992) reported EEG spindles when membrane potential of TC neurons is between −55 to −65 mV. Since RE nuclei project GABAergic inhibition to TC neurons, propofol, and volatile anesthetic potentiate GABAergic inhibition and probably concentration-dependently hyperpolarize the membrane potential of TC neurons. This might account for the transient increase in spindle activity when propofol Ce gradually increases. Because patients don't wake when spindle waves predominate, this is the level of anesthesia I maintain during clinical practice.

It is well-known that alpha oscillation (around 10 Hz) is observed when a person is calmly awake with eyes closed. This alpha oscillation is mostly derived from occipital regions (Guyton and Hall, 2011). By contrast, alpha rhythms observed during anesthesia or natural sleep are mostly derived from the frontal region. This alpha-rhythm shift has been characterized as anteriorization and can be seen in the findings of Purdon et al. (2013). Although, raw EEG is anesthetic-specific in detail, these concentration-related EEG changes commonly occur in anesthetics that potentiate *GABA*_A_ receptor (Hagihira, 2015).

To attenuate hemodynamic reactions at intubation, opioids such as fentanyl or remifentanil are usually also used during induction of anesthesia with propofol or thiopental. It is known that such opioids decrease propofol Ce at loss of response (LOR) to verbal command, something seen by Lysakowski et al. (2001) who, with the co-administration of opioids, however, also observed higher BIS™ values at LOR. Schraag et al. (2006) have reported that use of remifentanil significantly decreases propofol Ce at LOR, even though AEPex values, indices based on auditory evoked potential, remains high compared with patients who didn't receive remifentanil. Since BIS™ and AEPex values indicate level of brain activity, these results suggest that patients who received opioids along with anesthetic agents might still be conscious at LOR. At any rate, it isn't easy to truly judge whether a patient has lost consciousness or not, especially when opioids are co-administered.

## 4. EEG during rapid induction of anesthesia

Flaishon et al. (1997) have reported that BIS^*TM*^ transiently decreased to 20–30 after bolus administration of thiopental 4 *mg*·*kg*^−1^ or propofol 2 *mg*·*kg*^−1^. After the transient decrease, the index again increases, after which the patients regain consciousness. These changes in BIS™ index seem to be reliable indicators of level of consciousness. Observation of raw EEG, however, during rapid induction is quite specific. Just after loss of response owing to bolus administration of propofol or thiopental, large delta waves emerge. At that time the BIS™ index decreases to 20–30 (Figure 1I). Similarly, during slow induction by 8% sevoflurane, similar waveforms may also be transiently observed and the BIS™ index may decrease to 10–20 (Yamaguchi et al., 2003). Soon after that, alpha or theta waves predominate and BIS™ index increases to 30–50. During slow induction by sevoflurane, anesthetic concentration in the brain must increase monotonically. If this hold true, the changes in BIS™ index are odd. If BIS™ index were adequately indicating the level of hypnosis, the BIS™ would decrease monotonically. From this discrepancy, we have to wonder whether the algorithms in BIS™ are weighted toward indicating a low BIS™ index when large delta waves emerge. The plots seemed to be too well fitted to the putative level of hypnosis. Since the algorithms used for the calculation of BIS™ values have not been published, the facts cannot be verified.

Morimoto et al. (2004) have shown that when the suppression ratio (SR; the ratio of flat curve of EEG in the latest 1 min of EEG signal) is more that 40%, BIS™ is determined solely by SR according to the formula, index = (100-SR)*25/60. Even if the SR is zero, however, when large delta waves emerge, the BIS™ index value could decrease below 25. This observation is important because characteristic in some specific situations (Hagihira et al., 2004; Morimoto et al., 2005; Oda et al., 2006), the emergence of large delta waves makes it hard to discern the level of hypnosis from EEG.

## 5. EEG during recovery from anesthesia

When other factors have little influence, EEG changes following gradual administration of propofol are symmetrical during the course of induction and emergence from anesthesia (Figures 1F–H). The most clinically important moment is when the patient regains consciousness, or more practically, when there is response to verbal command. In clinical settings, timing seems to depend on residual anesthetic effect, wound pain or other stimuli, and the effects of analgesia from opioids, neural blockade, or other analgesic agents. When analgesia is insufficient, patients may regain consciousness even when residual anesthetic concentration is still high. In such cases, electromyogram (EMG) contamination is often observed. The findings in Hagihira et al. (2004) show that spindles may disappear when noxious stimuli is added. Of course, spindle pattern is not always predominant at an adequate surgical level of anesthesia; in fact, sufficiently anesthetized elderly patients often show low alpha power. While it is prudent, during maintenance of anesthesia, to suspect insufficient analgesia when spindle waves don't predominate, there may be other reasons for reduced spindle activity.

When analgesia is properly provided, patients normally wake up when amplitude on EEG decreases. Even so, anesthesiologists do occasionally encounter patients who don't respond to verbal commands even when the EEG patterns has returned to one normally corresponding wakefulness. This is more likely to occur when opioid Ce is still elevated, or neural blockade is in effect. As discussed in the previous section, this may be due to opioid-related reduction of propofol Ce at LOR to verbal command during induction of anesthesia. It is also known sensory blockade tends to decrease alertness, and this may also affect recovery from anesthesia. Anyway, during clinical practice it is not as easy to determine emergence from anesthesia as in the volunteer studies (Doufas et al., 2004; Iwakiri et al., 2005).

## 6. Summary

As discussed here, when anesthesia is gradually induced, EEG patterns during induction of and emergence from anesthesia seemed to mirror each other. Although, the data obtained from single channel EEG is rather limited, raw EEG information can still tell us much.

Finally, once we can reliably evaluate connectivity among several brain regions by multi-channel EEG monitoring (Ku et al., 2011; Lee et al., 2013), without stimulus testing, it may be possible to judge whether a patient is conscious or unconscious.

## Author contributions

The author confirms being the sole contributor of this work and approved it for publication.

### Conflict of interest statement

The author declares that the research was conducted in the absence of any commercial or financial relationships that could be construed as a potential conflict of interest.
